# The role of semantics in the perceptual organization of shape

**DOI:** 10.1038/s41598-020-79072-w

**Published:** 2020-12-17

**Authors:** Filipp Schmidt, Jasmin Kleis, Yaniv Morgenstern, Roland W. Fleming

**Affiliations:** 1 Justus Liebig University Giessen, Experimental Psychology, Otto-Behaghel-Str. 10F, 35394 Gießen, Germany; 2 Center for Mind, Brain and Behavior (CMBB), University of Marburgand Justus Liebig University, Giessen, Germany

**Keywords:** Psychology, Human behaviour, Cognitive neuroscience

## Abstract

Establishing correspondence between objects is fundamental for object constancy, similarity perception and identifying transformations. Previous studies measured point-to-point correspondence between objects before and after rigid and non-rigid shape transformations. However, we can also identify ‘similar parts’ on extremely different objects, such as butterflies and owls or lizards and whales. We measured point-to-point correspondence between such object pairs. In each trial, a dot was placed on the contour of one object, and participants had to place a dot on ‘the corresponding location’ of the other object. Responses show correspondence is established based on similarities between semantic parts (such as head, wings, or legs). We then measured correspondence between ambiguous objects with different labels (e.g., between ‘duck’ and ‘rabbit’ interpretations of the classic ambiguous figure). Despite identical geometries, correspondences were different across the interpretations, based on semantics (e.g., matching ‘Head’ to ‘Head’, ‘Tail’ to ‘Tail’). We present a zero-parameter model based on labeled semantic part data (obtained from a different group of participants) that well explains our data and outperforms an alternative model based on contour curvature. This demonstrates how we establish correspondence between very different objects by evaluating similarity between semantic parts, combining perceptual organization and cognitive processes.

## Introduction

**Figure 1:**
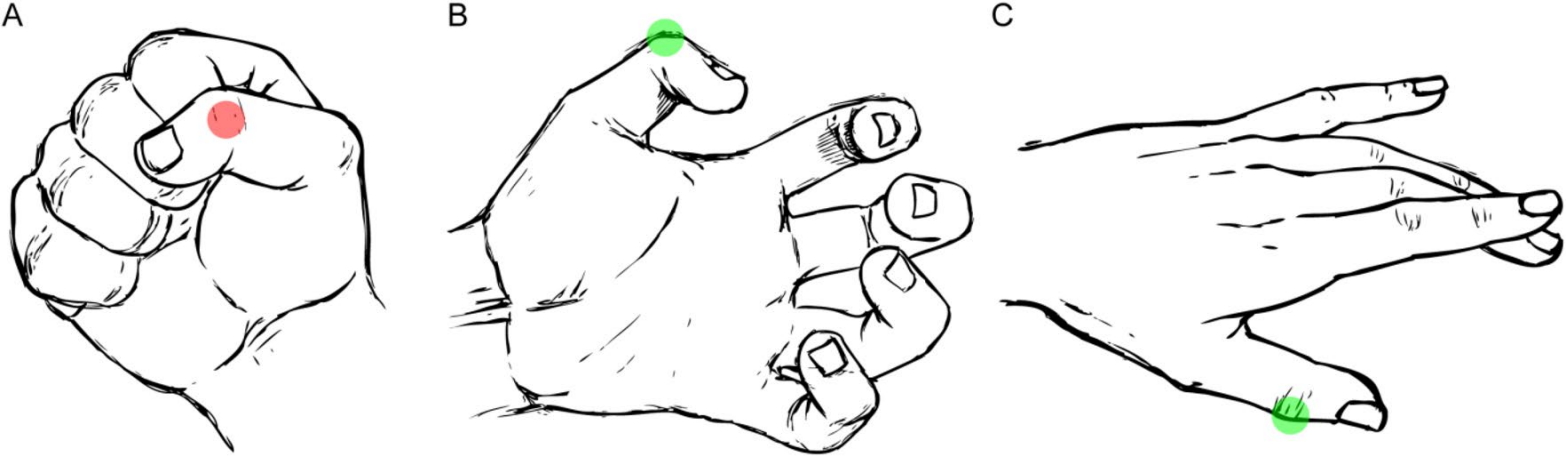
Correspondence problem and possible solutions. A major computational challenge is establishing correspondence between (**A**) and (**B**)–(**C**) across changes in viewpoint, object pose or non-rigid re-configuration. Observers generally have strong intuitions about the ‘correct’ solution, often with high agreement between observers. Drawings by Robert Marzullo (2017, https://ramstudioscomics.com).

We live in a world in which our survival depends critically on successful interactions with objects. This requires inferring an object’s properties—such as its material, potential usages, dangerousness and so on. We mostly infer these properties from our previous experiences about other objects from the same or similar class. For example, what we know about a peacock butterfly (e.g., fragile, able to fly, nectar eater, harmless), we can use to make inferences about other butterfly varieties. Only by constantly making such inferences, can we interact with objects in our environment without having to learn the properties of each newly encountered object *de novo*^1–5^. As object shape is arguably the most important cue for object recognition and concept learning (e.g.,^4,6–8^), shape presumably plays a major role in this generalization. In other words, we assume that peacock and lemon butterflies have similar properties because they have broadly similar shapes. Here, we consider a specific measure of the relationships between shapes: our striking ability to identify point-to-point correspondences between objects (Fig. 1;^9,10^).

Previous studies showed that perceived correspondence between object shapes is to some extent robust against changes in viewpoint and more complex transformations^11–17^. This is also true at the level of point-to-point correspondence, that is, when identifying the corresponding points on the surfaces of two objects (e.g.,^18–22^). In previous work, we found humans were very good at solving the point-to-point correspondence problem for 2D shapes across classes of rigid (e.g., rotation) and non-rigid transformations (e.g., growing new limbs)^9,10^, with high levels of agreement to the ground truth and other observers.

A simple heuristics model based on contour curvature, however, was better at predicting human responses than the ground-truth^9^. The heuristic model assumes observers identify salient locations on the original contour (e.g., a spike and a bump on an otherwise smooth contour) and then find the corresponding salient regions on the transformed contour (e.g., a spike and a bump on the rotated contour). Finally, observers establish point-to-point correspondence for intermediate locations on the shapes relative to these salient points. For example, if a particular location on the original contour is located halfway between the spike and the bump, they will choose as corresponding location that one halfway between spike and bump along the rotated object contour.

Such image-based heuristic approaches cannot, however, explain all point-to-point correspondences between objects. Shapes will often be very different making it near impossible to establish correspondence based on their geometrical features alone (e.g., curvature profiles). An alternative that we hypothesize here is that humans can also solve correspondence tasks by combining shape and semantic information. For example, in Fig. 1, it is hard to reconcile our intuitions about correspondences between the hands in A, B, and C with a correspondence based on geometrical features alone. Rather, we seem to use our knowledge about the semantic organization of the hand (‘the point lies on the knuckle of the thumb’) to guide our responses. Previous studies with unfamiliar objects show that correspondence can be established without such semantic information. In other words, semantics are not *necessary* for determining correspondence. Yet, it seems plausible that—if available—high-level semantic information *facilitates* spatial correspondence judgements. Indeed, here we test whether semantic cues are *sufficient* to override geometrical similarities between objects.

**Figure 2:**
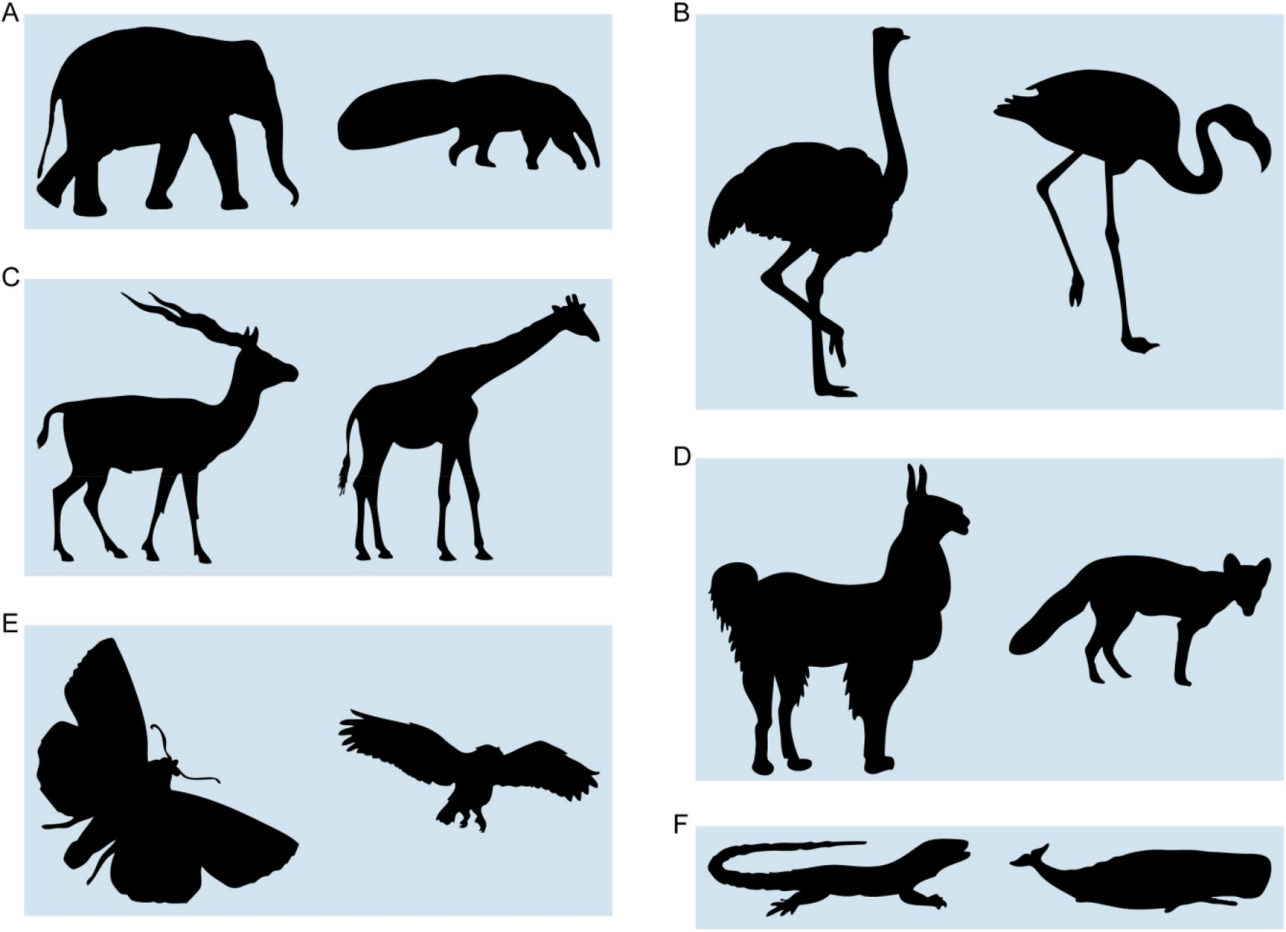
Stimuli of Experiment 1. Each pair was presented on the screen simultaneously, with the base shape to the left and the test shape to the right (arrangement the same as here in the figure). Images were obtained from different online databases and are reprinted with permission. (**A**) Elephant–Anteater (‘Elephant’ by depositphotos.com/bojanovic; ‘Anteater’ by depositphotos.com/160,377), (**B**) Ostrich–Flamingo (‘Ostrich’ by depositphotos.com/kaludov; ‘Flamingo’ by shutterstock/Yaroslavna Zemtsova), (**C**) Antelope–Giraffe (‘Antelope’ by shutterstock.com/Momo0607; ‘Giraffe’ by freedesignfile.com/Starder), (**D**) Lama–Fox (‘Lama’ by vecteezy.com; ‘Fox’ by shutterstock.com/Rey Kamensky), (**E**) Butterfly–Owl (‘Butterfly’ by shutterstock.com/ntnt; ‘Owl’ by yayimages.com/Perysty), and (**F**) Lizard–Whale (‘Lizard’ by shutterstock.com/angelp; ‘Whale’ by depositphotos.com/ktinte). All stimuli are available at 10.5281/zenodo.4304299.

It is well known that objects are not only perceived in terms of their overall shape, but also in terms of their parts^23–25^. Accordingly, observers might establish correspondence between very different shapes by relying on semantically labelled parts (e.g., the wings or legs of a butterfly). Specifically, we might segment objects into recognizable parts, such as legs, wings or tails, that can be matched across objects, and then use broadly the same heuristic as described above to interpolate between these sparse correspondences^9^. The key difference to previous work is that, rather than defining the salient regions that form the anchor points for correspondence by local geometrical features alone, those regions are instead defined by the semantic parts. This would allow observers to identify point-to-point contour correspondences even if contour shapes differ wildly. For example, if presented with an elephant and anteater (Fig. 2A), with geometrically very different outlines, observers would be able to match up the trunks of the two shapes and work out the correspondence between any given point based on its relative position along the trunk’s outline.

To test this hypothesis that humans establish correspondence between very different shapes based on the perceptual organization of the shapes together with previous knowledge about semantic parts, we obtained point-to-point correspondence judgments for contours with different shapes but similar part organizations (6 pairs of animal shapes, Experiment 1; Fig. 2) as well as for contours with the same shapes but different part organization (5 animal shapes with ambiguous interpretations, Experiment 2; Fig. 5). For the first set of contours (Experiment 1), it is very difficult to establish point-to-point correspondence based on shape features alone as shapes are very different. For the second set of contours (Experiment 2), it is impossible to use shape features at all as the contours are geometrically identical and differ only in their interpretation. Thus, Experiment 2 is designed to test the role of semantic part organization in establishing correspondence in the extreme. Specifically, by holding shape constant, the experiment tests whether semantics are sufficient to override purely geometrical factors in determining correspondence between shapes. Across both experiments, we can test to what extent observers agree in their correspondence responses under these challenging conditions, and whether we can explain their responses by a model based on part organization and semantic correspondence. For comparison, we contrast it with a simple model based on uniform sampling around the contour as well as with a model based on shape features.

Together with previous studies illustrating the role of shape features for correspondence between unfamiliar stimuli with no semantic part organization, this would show that depending on the available information human observers will flexibly rely on either perceptual or cognitive processes to establish correspondences. Specifically, in this paper we aim to demonstrate how we establish correspondence between very different objects by evaluating similarity between semantic parts, combining perceptual organization and cognitive processes.

## Experiments

### Experiment 1: Different geometry, similar parts

#### Participants

15 students from Justus-Liebig-University Giessen, Germany, with normal or corrected vision participated in the experiment for financial compensation (11 w, 4 m, mean age = 22.5 years, SD = 2.9). This number is based on our previous work using the same paradigm^10^. All participants gave informed consent, were debriefed after the experiment, and treated according to the ethical guidelines of the American Psychological Association. All testing procedures were approved by the ethics board at Justus-Liebig-University Giessen and were carried out in accordance with the Code of Ethics of the World Medical Association (Declaration of Helsinki).

#### Stimuli

Stimuli were 6 pairs of 2D contours (Fig. 2) that were chosen to have different shapes but similar part organization (e.g., same number of legs or wings). For each of the base shapes (left shapes in Fig. 2A–F), we defined 50 probe locations by sampling the contour at equidistant intervals starting from a random position on the contour.

#### Procedure

**Figure 3:**
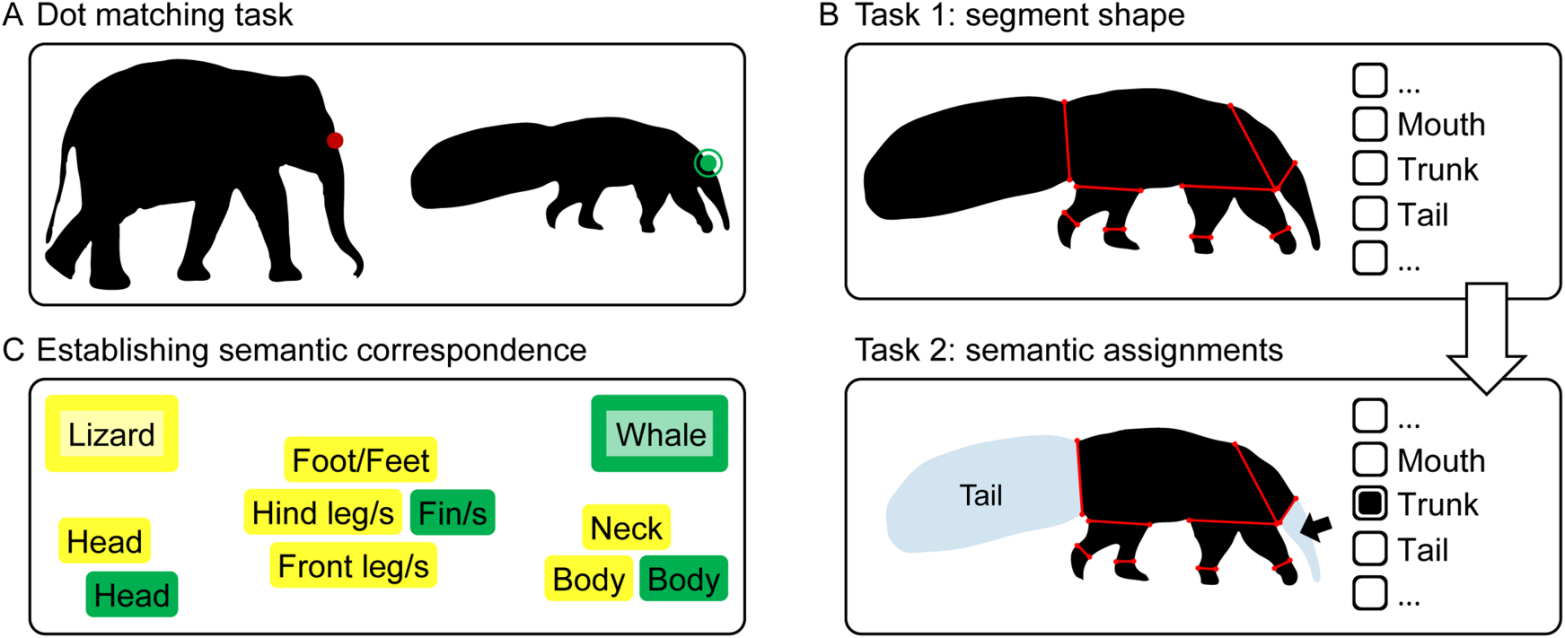
Overview of experimental paradigms. (**A**) Example response in the dot-matching task (Experiments 1 and 2), where participants see a probe point (red) on the contour of the base shape (e.g., elephant) and are instructed to place a bullseye (green) on the corresponding location on the contour of the test shape (e.g., anteater). (**B**) Example response for identifying and labeling parts (Experiment 3A). For each shape, participants first identify part cuts by using the computer’s mouse to choose two locations on the contour (Task 1). After they identified as many parts as they like to, they assign labels to these parts by selecting them and choosing a label from a list (Task 2). (**C**) Example response for establishing correspondence between semantic part labels (Experiment 3B). Participants sorted together color-coded names of animal parts identified in Experiment 3A. In the end, each part label of one animal has to be sorted together with a particular part label of the other animal. This includes cases in which several part labels are assigned to the same part label (e.g., neck and body of the lizard are both sorted together with the body of the whale).

Before the start of the experiment, participants were handed a written instruction with an outline of the experiment and the literal instruction ‘*Your task is to find the correspondence between the red dot on the left contour and the dot on the right contour. You can move a green dot with the mouse and confirm your selection with a mouse click. Please do not take any breaks while responding to a pair! You can take as much time for each decision as you need for a confident choice. Make sure to work thoroughly!*’ We also present the Elephant–Anteater pair as an example (Fig. 3A), with a red dot on the elephant contour and an unplaced green dot in the white space next to the anteater. In response to questions about how to perform the task, the experimenter replied that there was no right or wrong answer.

In each trial of the experiment, participants were presented with one of the shape pairs (Fig. 2A–F). The base shape (e.g., elephant) was presented on the left side and the test shape (e.g., anteater) on the right side of the screen. We successively presented probe points on the contour of the base shape and asked participants to use the mouse to place a small bullseye marker ‘at the corresponding location’ on the contour of the test shape (Fig. 3A). The probe point was a small red dot (0.10° radius) and the bullseye was a small green dot (0.10° radius) surrounded by a ring (0.75° radius). After participants confirmed their choice with a mouse click, the probe point was replaced by a probe at a different location, where the location was selected across 50 preselected locations. In addition to presenting the points one by one, the locations were also presented in a random order to minimize the influence of a participant’s previous decision from nearby locations. Each pair of shapes remained on screen until participants responded to all 50 probe points, enabling a dense mapping of perceived correspondences. Each participant responded to each of the stimulus pairs and to the same probe points; across participants pairs were presented in random order. Finally, base and test shapes were either presented in the same orientation (as in Fig. 2) or in different orientation (e.g., elephant and anteater facing each other), with orientation counterbalanced across pairs and participants.

Stimuli were presented on a white background on a EIZO CG277 monitor at a resolution of 2560 × 1440 pixels and a monitor refresh rate of 59 Hz, controlled by MATLAB2018a (The MathWorks, Inc., Natick, Massachusetts, United States) using the Psychophysics Toolbox extension^26^. The two shapes of each pair were uniformly scaled so that their bounding boxes had the same area. The width of the resulting shapes varied between 15.33° and 22.99° of visual angle, and the height varied between 4.42° and 27.81° of visual angle (with a distance between participants and monitor of about 50 cm).

#### Analysis

Note that for our stimuli there is no ground truth or mathematically correct solution to map the base to the test shape. Consequently, we analyze the results with respect to the extent to which participants agree with each other, and test how well this agreement can be explained by different models (random, shape-based and semantic-based models).

#### Results and discussion

**Figure 4:**
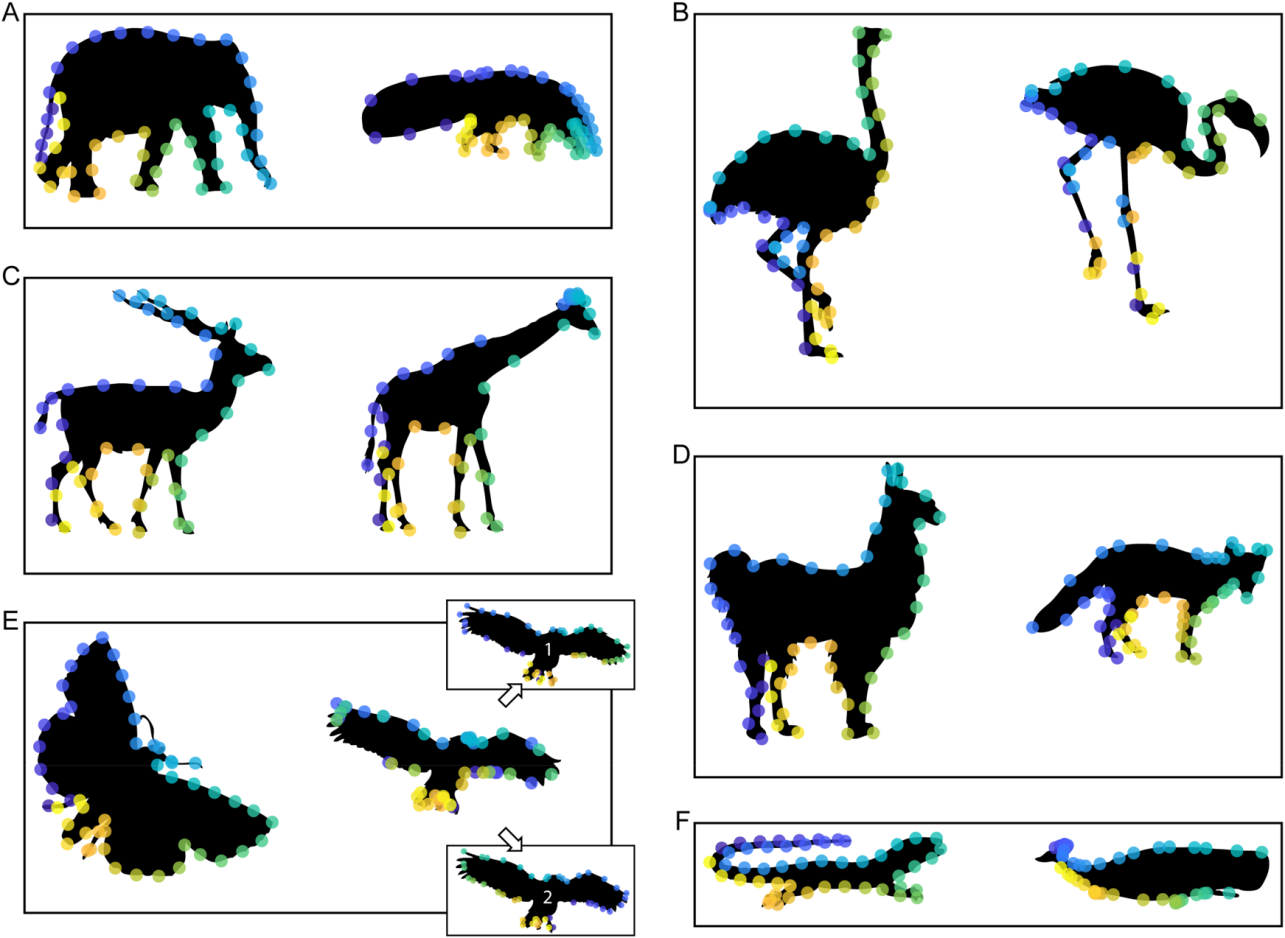
Overview of results of Experiment 1. On each base shape (left, e.g., elephant), we plot the 50 equidistant probe points. On each test shape (right, e.g., anteater), we plot the corresponding participant responses for each of the points. Participant responses are summarized by determining the median position along the length of the contour. (**A**–**D**) For most pairs and probe points, the order of points on the test shapes is the same as that on the base shape even though probe points were queried one at a time. (**E**) For the Butterfly–Owl pair, we also show results separately for participants arranging their responses in the same (inset 1) or reversed order (inset 2) as the probe points. Data are available at 10.5281/zenodo.4304299.

Results of Experiment 1 are plotted in Fig. 4. Responses of participants are highly systematic with generally (i) high agreement between participants, (ii) well-preserved ordering of corresponding locations, and (iii) corresponding locations on similar semantic parts of base and test shape (e.g., probe points on the elephant’s trunk are matched with locations on the anteater’s snout; see Fig. 4A and Sect. Modeling).

With respect to (i), we quantify agreement between participants as response congruity on a scale from 0, indicating congruity of random responses, up to 1, indicating perfect congruity. Specifically, for each probe location on the base shape, we calculated the average of distances between all participants’ responses along the contour of the test shape (distances expressed as a percentage of test shape perimeter). Thus, congruity refers to the spatial proximity between all responses from the same probe point. We calculate the grand mean of these average distances for all probe points, obtaining a single congruity score for each pair of shapes. Finally, we project that score onto a continuum between random and perfect congruity by subtracting 1 from the ratio of mean of distances to randomly placed responses on the test shape, where a value of 1 refers to perfect congruity with all responses at the very same location (i.e., zero distance between responses of different participants). The results showed that for all pairs, participants are significantly more congruent than the random model (pairs A–F: 0.90, 0.86, 0.90, 0.88, 0.48, 0.71; Wilcoxon signed rank test: − 5.61 < Z <  − 6.15, all *p* < 0.001).

Why are participants considerably less consistent for the Butterfly–Owl pair (0.48) than for the other tested pairs? We reasoned that this resulted from the ambiguous 3D orientation that allows seeing the animals as viewed from the front or back, rendering correspondence ambiguous too. In line with this idea, individual participants either arranged their responses on the owl in the same (n = 8; median responses of this group: inset 1 in Fig. 4E) or reversed order (n = 7; inset 2 in Fig. 4E) as the probe points on the butterfly, with congruity of 0.76 and 0.81 within both groups. This pattern of responses might be to some extent explained by the presentation of butterfly and owl either heading in the same direction (as depicted in Fig. 2E) or in different directions. Indeed, the majority of participants presented with butterfly and owl heading in the same direction also arranged responses in the same order (5 out of 7), while different heading directions rather tended to produce reversed orderings (5 out of 8).

With respect to (ii), we quantify the extent to which responses preserved the ordering points on the test shape. For this, we calculated how often the ordering of the median responses reversed (i.e., how often a given dot was flanked by different dots on the test shape than on the base shape). We compared the number of reversals with the mean number of reversals that occur with the same number of random responses. The results of this analysis revealed that ordering was preserved significantly more in the participants’ median responses than in the random model (preserved order in pairs A–F: 100%, 80%, 94%, 92%, 16%, 62%; Wilcoxon signed rank test: − 2.23 < Z <  − 6.94, *p* < 0.026). Again, the preservation of ordering is much higher for the Butterfly–Owl pair when considering the two groups separately based on their perceived orientation (Fig. 4E, inset 1: 56%; inset 2: 68%). Regarding (iii), a formal quantification of corresponding locations on similar semantic parts is presented in the Results and Discussion of Experiment 3.

Together, these analyses provide an initial indication that participants can consistently identify correspondences across quite widely divergent shapes. This consistency between participants in establishing correspondences might be explained by common strategies based on contour curvature^9^ or based on semantic part organization (e.g., elephant’s trunk and anteater’s snout). In Experiment 2, we sought to further test to what extent semantic part organization can be used to establish correspondence, by testing stimulus pairs of ambiguous shapes that were physically identical but could be interpreted with different semantic part organizations.

### Experiment 2: Identical geometry, similar parts

#### Participants

15 students from Justus-Liebig-University Giessen, Germany, with normal or corrected vision participated in the experiment for financial compensation (12 w, 3 m, mean age = 23.7 years, SD = 4.1). Again, the number of participants is based on our previous work^10^. All other details and participant procedures were the same as in Experiment 1.

#### Stimuli

**Figure 5:**
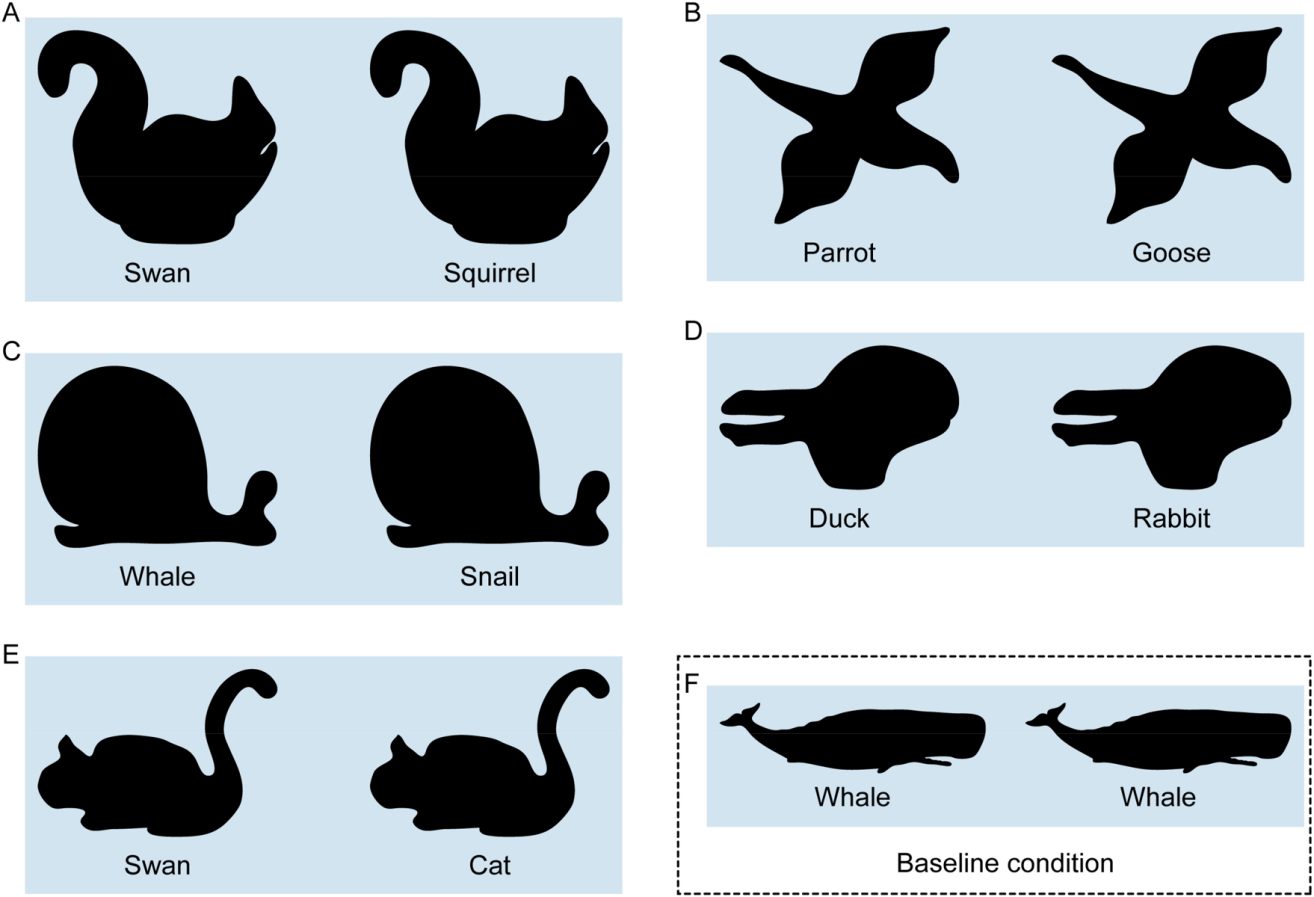
Stimuli of Experiment 2. Each pair was presented on the screen simultaneously, with the base shape to the left and the test shape to the right (arrangement the same as here in the figure). Images were drawings by author J. K. inspired by or copying stimuli from previous papers showing ambiguity in visual perception. (**A**) Swan–Squirrel from Fisher^27^, (**B**) Parrot–Goose by Tinbergen^28^, (**C**) Whale–Snail by Bernstein and Cooper^29^, (**D**) Duck–Rabbit by Jastrow^30^, and (**E**) Swan–Cat by Bernstein and Cooper^29^. (**F**) Example of the baseline condition with the same shape and label. All stimuli are available at 10.5281/zenodo.4304299.

Stimuli were 5 pairs of shapes (Fig. 5A–E) that were chosen to have the same shapes but different part organization. Specifically, we used ambiguous figures to disentangle shape from part organization. For example, the Swan–Squirrel (Fig. 4A) can be seen either as a swimming swan oriented to the left, or a crouching squirrel oriented to the right. To measure baseline performance, we also added a condition with the same shape and the same label for two additional shapes: the whale (Fig. 5F) and the antelope (Fig. 4C) from Experiment 1. In the interest of keeping participant sessions below an hour, the data for the second shape (antelope) was obtained from a different set of participants that took part in a separate experiment (n = 15, 7 w, 8 m, mean age = 24.5 years, SD = 3.1).

#### Procedure

The written instruction was the same as in Experiment 1 with the Duck–Rabbit pair as an example, with a red dot on the duck contour and an unplaced green dot in the white space next to the rabbit (both with semantic labels presented below the shape).

Also, the procedure was the same as in Experiment 1, again with semantic labels presented below each shape (different labels for the ambiguous stimuli, Fig. 5A–E, identical labels for the stimuli that we use as baseline condition, Fig. 5F).

Presentation details were the same as in Experiment 1; after scaling, the width of the resulting shapes varied between 15.44° and 28.22° of visual angle, and the height varied between 11.39° and 13.63° of visual angle (with a distance between participants and monitor of about 50 cm).

#### Results and discussion

**Figure 6:**
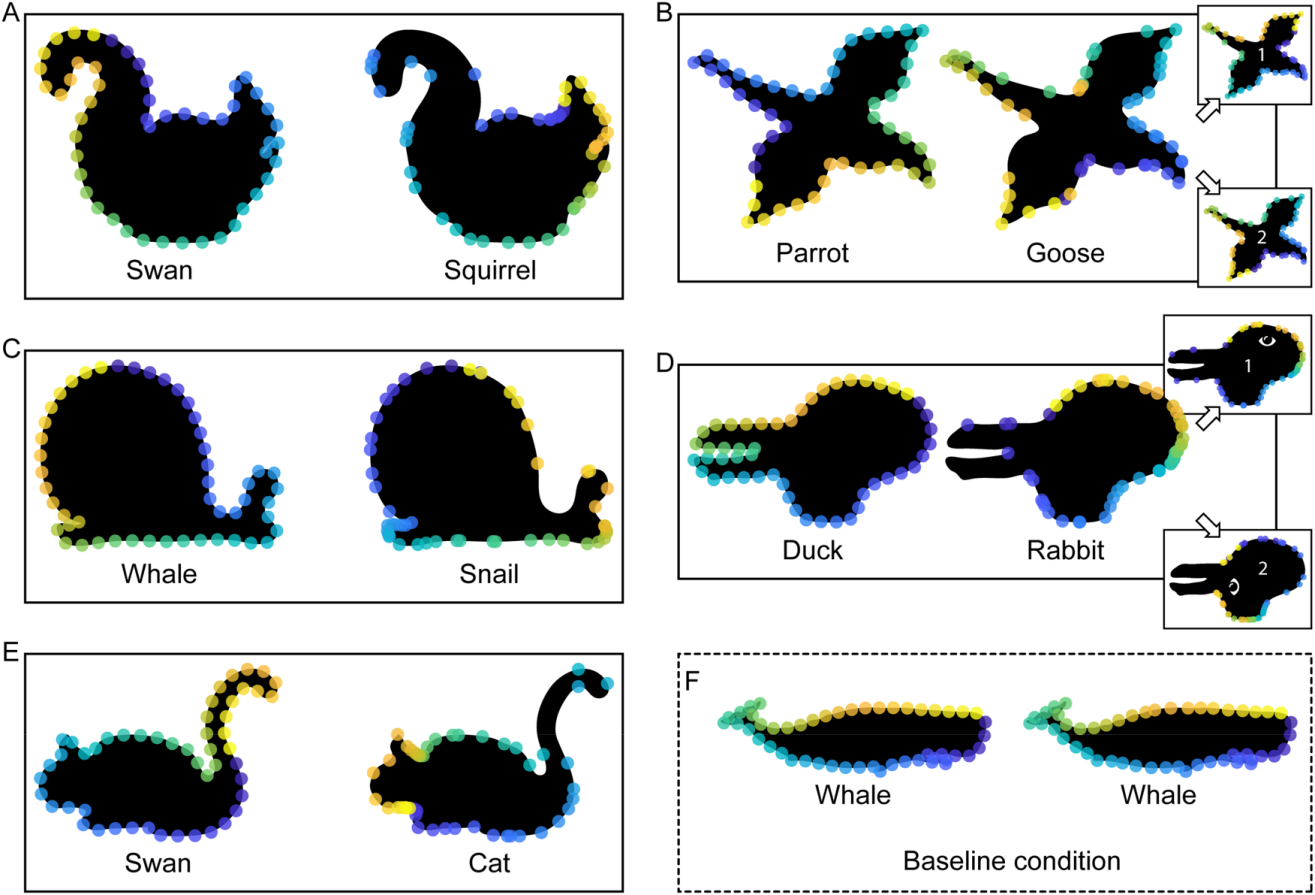
Overview of results of Experiment 2. On each base shape (left, e.g., swan), we plot the 50 equidistant probe points. On each test shape (right, e.g., squirrel), we plot the corresponding participant responses for each of the points. Participant responses are summarized by determining the median position along the length of the contour. (**A**,**C**,**E**,**F**) For most pairs and probe points, the order of points on the test shapes is the same as that on the base shape even though probe points were queried one at a time. (**B**) For the Parrot–Goose pair, we also show results separately for participants arranging their responses in the same (inset 1) or reversed order (inset 2) as the probe points. (**D**) For the Duck–Rabbit pair, we also show results separately for participants interpreting the rabbit as looking to the right (inset 1) or looking down (inset 2) (eyes added for illustration purposes). Data are available at 10.5281/zenodo.4304299.

Figure 6 shows results of Experiment 2. Again, responses of participants are highly systematic; however, they are somewhat lower with respect to the (i) agreement between participants, (ii) preserved ordering of corresponding locations, but still show (iii) corresponding locations on similar semantic parts of base and test shape (e.g., probe points on the swan’s head are matched with locations on the squirrel’s head; Fig. 6A).

With respect to (i), we quantify agreement between participants as in Experiment 1; participants are less congruent than in Experiment 1 but still more than predicted by the random model (pairs A–E: 0.75, 0.28, 0.40, 0.37, 0.76; Wilcoxon signed rank test: − 5.85 < Z <  − 6.16, all *p* < 0.001). We suggest that the lower congruency compared to Experiment 1 can be explained by two factors. First, the semantic part organization was less clear for ambiguous shapes of Experiment 2 compared to unambiguous shapes of Experiment 1—as unambiguous shapes have more contour details and are more prototypical contours of animals. Second, the correspondence between semantic parts was less clear for ambiguous shapes of Experiment 2—as part organization (i.e., the viewpoint and pose of the objects) of unambiguous base and test shapes in Experiment were more similar (with exception of pair Lizard–Whale).

Again, 3D orientation is ambiguous for the parrot and goose and, indeed, individual participants either arranged their responses on the goose in the same (n = 5; median responses of this group: inset 1 in Fig. 6B) or reversed order (n = 10; inset 2 in Fig. 6B) as the probe points on the parrot, with congruity of 0.86 and 0.51 within both groups. Again, this corresponds to some extent to observers’ perception of the same (cf. Figure 5B) or different orientation of parrot and goose: the majority of participants presented with both heading in the same direction also arranged responses in the same order (n = 5 out of 8), while different heading directions produced reversed orderings (n = 7 out of 7).

Interestingly, some participants also reported having interpreted the rabbit (Fig. 6D) differently from the classical interpretation: while most participants reported seeing a rabbit looking to the left or right (n = 12; see illustration and median responses: inset 1 in Fig. 6D), a few participants reported to see a rabbit looking down (n = 3; see illustration and median responses in inset 2 in Fig. 6D), with congruencies of 0.50 and 0.70 within these groups. This was rather not explained by different heading directions as half of participants seeing the rabbit as looking to the left or right (n = 6) were presented with both heading in the same direction and the other half (n = 6) with different directions.

With respect to (ii), we quantify the preservation of ordering as in Experiment 1, showing that ordering was preserved significantly more in the participants’ median responses than in the random model (preserved ordering in pairs A–E: 82%, 90%, 82%, 58%, 82%; Wilcoxon signed rank test: − 2.23 < Z <  − 6.94, *p* < 0.026). The preservation of ordering is similar for the Parrot–Goose pair (‘1’: 88%; ‘2’: 94%) and the Duck–Rabbit pair (‘1’: 56%; ‘2’: 68%) when considering the two groups separated by their perceived orientation.

As for Experiment 1, regarding (iii) we present a formal quantification of corresponding locations on similar semantic parts in the Results and Discussion of Experiment 3. However, in contrast to Experiment 1, the effect of semantic interpretation is already evident from the fact that participants’ responses for the test shape are not identical to the base shape—as semantics were the only difference between the two.

These analyses indicate that even identical shapes yield different correspondences when they have a different semantic part organization. Taken together, Experiments 1 and 2 suggest that semantic features affect how corresponding points are placed. To test this hypothesis directly, we conducted a third experiment to obtain local semantic labels for the stimuli in Experiments 1 and 2, using a part segmentation and labeling task, and subsequently tested different models to explain human correspondence responses.

### Experiment 3: Semantic labeling of part structures

In Experiment 3, we identified parts and semantic part labels for all shapes of Experiments 1 and 2. In Experiment 3A, participants identified and labeled parts; in Experiment 3B, participants established correspondence between semantic part labels. We used this information to build a model based on semantic part organization for predicting participants’ responses in Experiments 1 and 2.

#### Experiments 3A and 3B: Participants

21 students from Justus-Liebig-University Giessen, Germany, with normal or corrected vision participated in the experiments for financial compensation. A group of 12 students (7 w, 5 m, mean age = 23.7 years, SD = 3.8) participated in Experiment 3A (Identifying and labeling parts) and a group of 9 students (6 w, 3 m, mean age = 24.2 years, SD = 2.5) participated in Experiment 3B (Correspondence between semantic part labels). All other details and participant procedures were the same as in Experiment 1.

#### Experiment 3A: Stimuli

Stimuli were all individual shapes from Experiments 1 and 2.

#### Experiment 3A: Procedure

In each trial, participants were presented with one of the shapes together with 16 labels on the right side of the screen: ‘Headʼ, ‘Bodyʼ, ‘Eye/sʼ, ‘Neckʼ, ‘Front leg/sʼ, ‘Hind leg/sʼ, ‘Foot/Feetʼ, ‘Ear/sʼ, ‘Trunkʼ, ‘Mouthʼ, ‘Antennaʼ, ‘Horn/sʼ, ‘Beakʼ, ‘Wing/sʼ, ‘Tailʼ, ‘Fin/sʼ, and ‘None of theseʼ (Fig. 3B). Using the point and click operations of the computer’s mouse, participants completed two tasks for each shape. In the first task (inspired by^31^), they defined part boundaries by selecting two locations on the contour (with the restriction that the resulting boundary would not intersect existing boundaries or the contour). After defining as many parts as they wanted, participants proceeded to the second task where they assigned labels by selecting each part in sequence and choosing a label from those on the right side of the screen. Each part was assigned only one label, but labels could be used for more than one part. After labelling all of the parts, participants continued with the next shape. Each participant responded to each of the shapes, presented in random order. Presentation details and size of stimuli were the same as in Experiments 1 and 2.

#### Experiment 3A: Results

To determine to which extent participants thought that the provided labels were not sufficient to name the parts, we calculated the average percentage of contour sections labelled ‘None of theseʼ. As these percentages were very low (Experiment 1: 0.12%; Experiment 2: 3.64%), we assume that participants considered the labels sufficient to name the great majority of contour parts.

**Figure 7:**
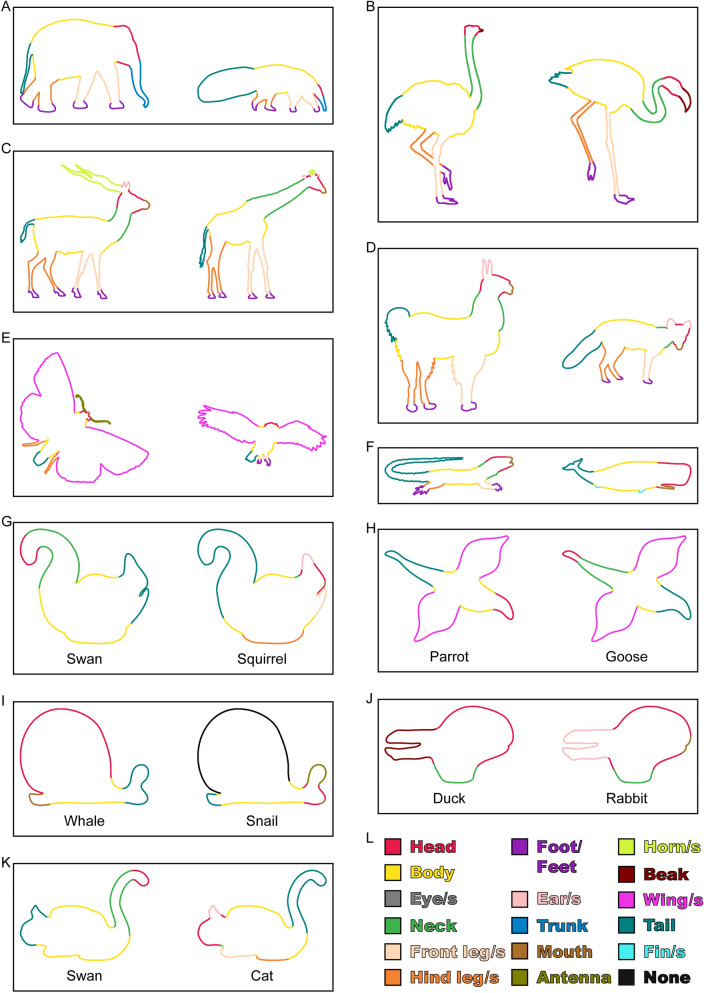
Overview of results of Experiment 3A. (**A**–**J**) On each shape, we plot for each point on the contour the most frequent label assigned to that point. (**L**) Labels and colors. Data are available at 10.5281/zenodo.4304299.

Then, we identified the most frequent label for each point on the contour for each individual shape. For this, we counted for each point on the contour the frequency (i.e., the number of participants) with which each label was assigned to that point. Then, we assigned the most frequent label to that point (Fig. 7).

#### Experiment 3B: Stimuli

Stimuli were printed paper cards, with one card for each of the most frequent labels obtained in Experiment 3A. Cards were color-coded by base shape (yellow) and test shape (green) (Fig. 3C).

#### Experiment 3B: Procedure

Participants were handed all cards belonging to a pair of shapes (e.g., all most frequent part labels for ‘Elephantʼ on yellow cards, and all most frequent part labels for ‘Anteaterʼ on green cards), together with two cards to identify which color belonged to which animal (e.g., a yellow ‘Elephantʼ and green ‘Anteaterʼ card; Fig. 3C). No visual shapes were presented, only words (e.g., for ‘Elephantʼ most frequent labels were ‘Headʼ, ‘Bodyʼ, ‘Front leg/sʼ, ‘Hind leg/sʼ, ‘Foot/Feetʼ, ‘Trunkʼ, and ‘Tailʼ, and exactly the same labels for ‘Anteaterʼ; see Fig. 7A). Then, participants were asked to sort cards together so that each part label of one shape was assigned to a particular label of the other shape; they also were allowed many-to-one but not many-to-many assignments (e.g., antenna and head of the butterfly might both be sorted together with the head of the owl but could not also be sorted with its wing; Fig. 7E). Note that for most pairs this was a trivial task as base and test shape were described by the very same set of semantic labels (as for Elephant–Anteater; Fig. 7). Each participant sorted together the part labels for all of the pairs, successively in random order.

#### Experiment 3B: Results

For each pair of shapes and each part label, we identified the most frequent correspondences (i.e., the labels sorted together by the most participants). This provides us with one-to-one correspondences between parts, based on semantic information alone. Consequently, we can build a model based on semantic part organization that can also predict participantsʼ responses for parts with non-identical labels (e.g., most participants sorted together the beak of the duck and the mouth of the rabbit; Fig. 7J). Data are available at 10.5281/zenodo.4304299.

## Modeling

In this section, we present our *semantic organization model* to predict human correspondence responses, and compare it to plausible alternative models. Note that the model is not purely image-computable: it relies on semantic part labels derived from participant data. However, the model does provide quantitative predictions at finer spatial scale (position of corresponding points) than the raw data from which the predictions are derived (semantic label data from Experiment 3).

For comparisons between model predictions and human responses we report T-tests and, as a measure of effect size, corresponding Scaled JZS Bayes factors (*BF*_10_), using a Jeffrey-Zellner-Siow Prior (Cauchy distribution on effect size) with a default scale factor of 0.707^32^. *BF*_10_ expresses the probability of the data given H1 relative to H0 (i.e., *BF*_10_ > 1 is in favor of H1). *BF*_10_ > 3 can be considered as ‘some evidence,’ *BF*_10_ > 10 as ‘strong evidence,’ and *BF*_10_ > 30 as ‘very strong evidence’ for H1, whereas *BF*_10_ < 0.33 can be considered as ‘some evidence,’ *BF*_10_ < 0.1 as ‘strong evidence,’ and *BF*_10_ < 0.03 as ‘very strong evidence’ for H0^33^.

### Semantic organization model

**Figure 8:**
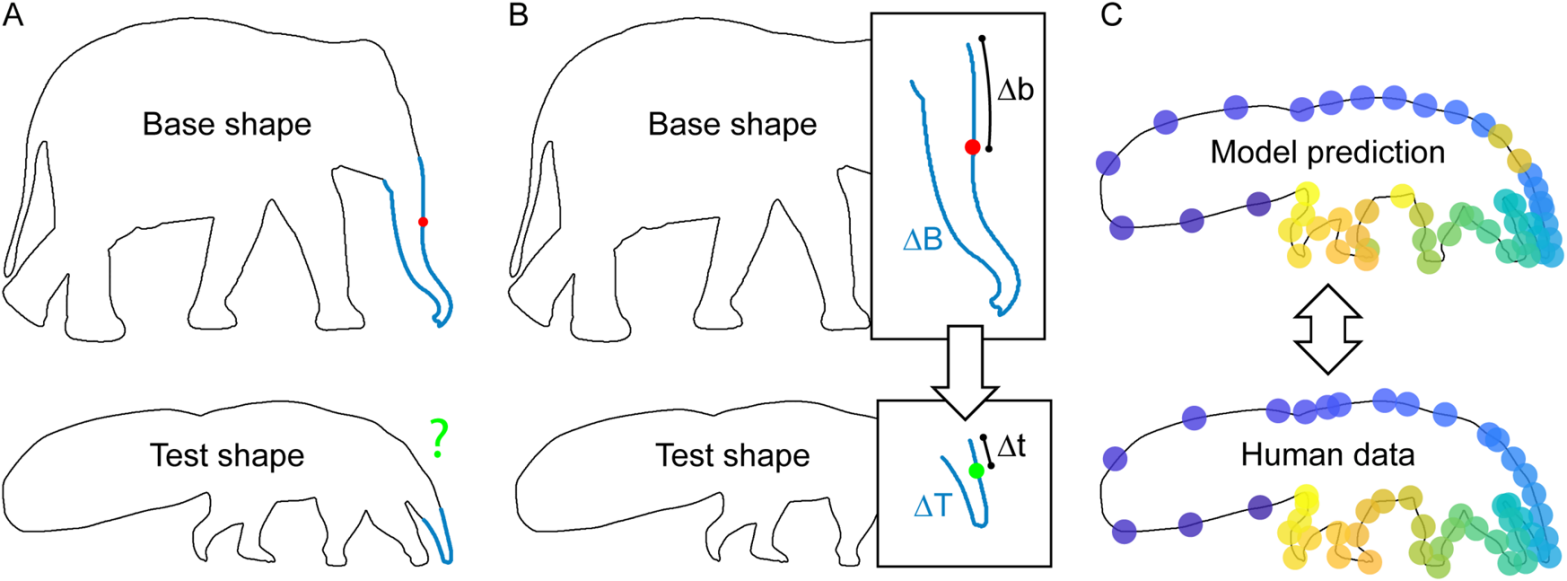
Illustration of our extended model. The model is based on the identified parts, their semantic labels and the semantic correspondences from Experiment 3. (**A**) First, for each probe point, we identify the part on the base shape it’s located on. Second, we find the corresponding semantic part on the test shape (using labels and semantic correspondences). (**B**) Third, we define the position of the probe point relative to the start and end of the semantic part by calculating its proportion of the length of the semantic part contour (*p* = ∆b/∆B) along the heading direction (as determined by the similarity in ordering of corresponding semantic parts). Fourth, we predict the test point by using that proportion to define its position relative to the start and end of the corresponding semantic part on the test shape (∆t = ∆T*p). (**C**) From this we obtain a singular predicted location on the test shape for each probe point. By comparing that prediction to the median human response and averaging across all points, we get a prediction accuracy score for each pair of shapes.

Our previous work^9^ suggested that the visual system identifies and establishes correspondence for a few salient landmarks on the shape, and infers the position of other points on the contour relative to these. This provides a robust method to infer correspondence but is only possible if shapes are similar enough to enable correspondence between the landmarks to be established. Here, we extend this model by suggesting that this correspondence can also be established based on semantic part organization. If correspondence for salient landmarks is difficult or not possible to establish, observers might refer to semantic part organization to establish point-to-point correspondence. In line with our previous model^9^, they would infer the position of points relative to identified semantic correspondences. Specifically, the semantic organization model (Fig. 8) generates predictions for each probe point on the base shape by identifying its location on a semantic part (e.g., on the Elephant’s trunk) and finding the same relative position on the corresponding semantic part of the test shape (e.g., on the Anteater’s snout). By this, we can compare the predicted locations to the median human responses for every point that we tested.

**Figure 9:**
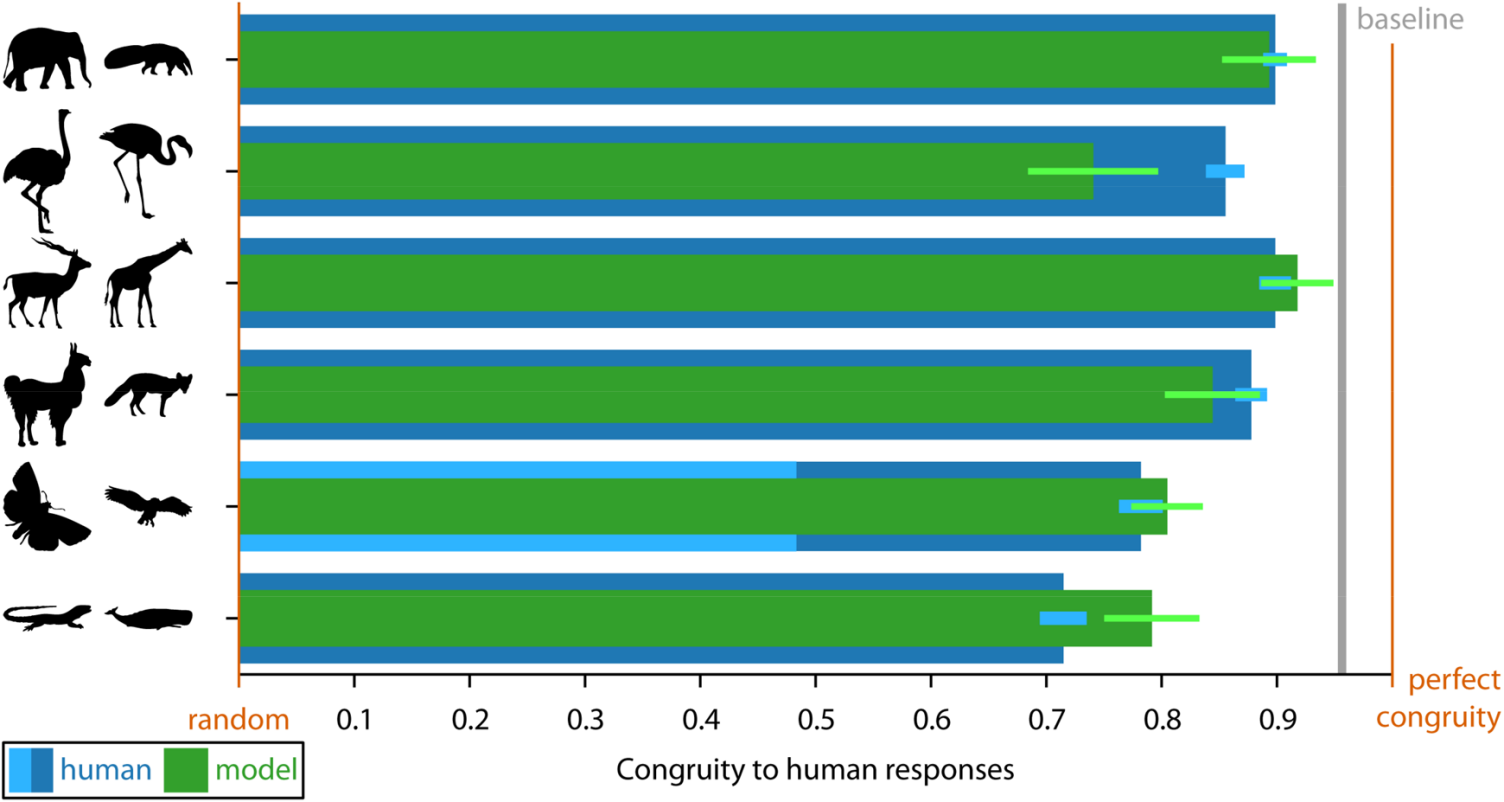
Human results and semantic organization model for Experiment 1. We plot response congruity on a continuum between random (0.0: congruity between responses of random model) and perfect congruity to human responses (1.0: all responses on top of each other). Horizontal bars plot congruity and standard errors for human responses (dark blue) and for the semantic organization model (green), separately for each pair of shapes. For the Butterfly–Owl pair, we also report congruity when measured across the two response types (light blue bar, see text for explanation). Finally, we plot the congruity of human responses in the baseline condition with the two pairs of identical shapes (Whale–Whale and Antelope–Antelope) as reference (grey bar).

An overview of the results of Experiment 1 is presented in Fig. 9, with human responses and predictions from the semantic organization model plotted with respect to their congruity with (other) human responses between random and perfect congruity (for details on calculating congruity see Results and discussion of Experiment 1). The figure shows that congruity between participants is generally high and much closer to perfect congruity than to the congruity of random responses (blue horizontal bars; Fig. 9). Also, when calculating the congruity between the predictions of the semantic organization model and human responses, we see that the model is often well in the range of human responses (green horizontal bars; Fig. 9): in other words, it often explains human responses as well as other human responses. This suggests that the model is capturing the relevant aspects of observed human response behavior.

For statistical testing, we calculated the distances of human responses (and model predictions) to the median human response for each pair of shapes and sample points, expressed as a percentage of contour perimeter^9,10^. The average distances for human responses to the median responses for each pair of shapes were 2.6% (Elephant–Anteater), 3.6% (Ostrich–Flamingo), 2.5% (Antelope–Giraffe), 5.5% (Butterfly–Owl; across two types: 13.0%), and 7.2% (Lizard–Whale) (Table 1; grand average: Fig. 12). The average distances for the semantic organization model predictions were 2.7%, 6.5%, 2.1%, 3.9%, 4.9% (12.1%), and 5.2% (Table 1; grand average: Fig. 12). T-tests for each pair (across the 50 sample points) showed no significant difference between the distances of human results and model predictions to median responses (Table 1).

**Table 1:** Overview of modeling results and statistics for Experiment 1.

Model	Pair	Human–human distance	Model-human distance	T(49)	*p*	*BF*_10_
Semantic organization	Elephant–Anteater	2.6	2.7	− 0.12	.905	0.21
	Ostrich–Flamingo	3.6	6.5	− 1.94	.058	1.11
	Antelope–Giraffe	2.5	2.1	0.63	.533	0.25
	Lama–Fox	3.0	3.9	− 0.81	.421	0.28
	Butterfly–Owl	5.5 (13.0)	4.9 (12.1)	0.59	.561	0.25
	Lizard–Whale	7.2	5.2	1.60	.118	0.65
Uniform sampling	Elephant–Anteater	2.6	8.8	− 6.34	< .001*	> 30
	Ostrich–Flamingo	3.6	14.8	− 7.52	< .001*	> 30
	Antelope–Giraffe	2.5	3.8	− 1.93	.059	1.09
	Lama–Fox	3.0	7.7	− 5.79	< .001*	> 30
	Butterfly–Owl	5.5 (13.0)	5.2 (13.6)	0.25	.805	0.22
	Lizard–Whale	7.2	8.9	− 1.61	.114	0.66
Curvature-based	Elephant–Anteater	2.6	1.4	− 2.82	.007*	6.64
	Ostrich–Flamingo	3.6	16.7	− 6.49	< .001*	> 30
	Antelope–Giraffe	2.5	6.2	− 3.01	.004*	10.59
	Lama–Fox	3.0	3.0	0.02	.981	0.21
	Butterfly–Owl	5.5 (13.0)	8.9 (17.0)	− 2.66	.011	4.60
	Lizard–Whale	7.2	15.0	− 4.00	< .001*	> 30
Combined semantic and curvature-based	Elephant–Anteater	2.6	3.1	− 1.69	.097	0.75
	Ostrich–Flamingo	3.6	6.5	1.00	.322	0.33
	Antelope–Giraffe	2.5	2.2	− 0.31	.755	0.22
	Lama–Fox	3.0	4.0	− 1.42	.162	0.52
	Butterfly–Owl	5.5 (13.0)	4.9 (12.1)	NaN	NaN	NaN
	Lizard–Whale	7.2	4.9	3.01	.004*	10.52

Significant *p* values are marked with an asterisk (Bonferroni-corrected significance level *p* < .008). Note that the combined semantic and curvature-based model is tested against the semantic organization model rather than against human data (NaNs follow from exactly the same values for both models).

**Figure 10:**
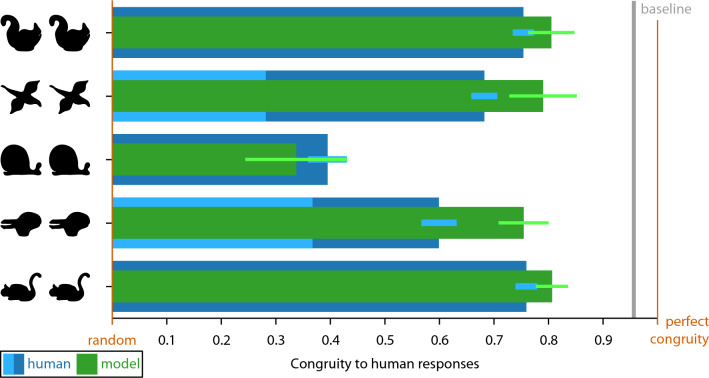
Human results and semantic organization model for Experiment 2. We plot response congruity on a continuum between random (0.0: congruity between responses of random model) and perfect congruity to human responses (1.0: all responses on top of each other). For the Parrot–Goose pair, we also report congruity when measured across the two response types (light blue bar, see text for explanation). For the Duck–Rabbit pair, we also report congruity when measured across the two semantic interpretations (light blue bar, see text for explanation). For other details see Fig. 9.

For Experiment 2, we again see that congruity is generally closer to perfect congruity than to the congruity of random responses (exception: Whale–Snail; blue horizontal bars; Fig. 10). However, it is also significantly lower than in Experiment 1. Before generating predictions from the semantic organization model, we inversed the order of contour points, as the two interpretations of each shape (e.g., swan and squirrel) were always heading different ways (e.g., swan to the left and squirrel to the right; Fig. 5A). When calculating the congruity between the resulting model predictions and all individual human responses, we again see that even though congruity is generally lower than in Experiment 1, the pattern of human responses is well replicated in the predictions (green horizontal bars in Fig. 10), again suggesting that the semantic organization model is a good model of participant behavior.

For statistical testing, we again calculated average distances to the median responses. For human responses, distances for each pair of shapes were 6.2% (Swan–Squirrel), 7.9% (Parrot–Goose; across two types: 18.0%), 15.0% (Whale–Snail), 10.0% (Duck–Rabbit; across two types: 15.8%), and 6.0% (Swan–Cat) (Table 2; grand average: Fig. 12). For the semantic organization model predictions, they were 4.9%, 5.3% (6.8%), 16.5%, 6.2% (6.6%), and 4.8% (Table 2; grand average: Fig. 12). When comparing the distances of human results and model predictions to median responses, they were only significantly different for one (Duck–Rabbit) but not for the other four pairs (Table 2).

**Table 2:** Overview of modeling results and statistics for Experiment 2.

Model	Pair	Human–human distance	Model-human distance	T(49)	*p*	*BF*_10_
Semantic organization	Swan–Squirrel	6.2	4.9	1.18	.243	0.39
	Parrot–Goose	7.9 (18.0)	5.3 (6.8)	1.61	.114	0.66
	Whale–Snail	15.0	16.5	− 0.55	.602	0.24
	Duck–Rabbit	10.0 (15.8)	6.2 (6.6)	2.85	.006*	7.20
	Swan–Cat	6.0	4.8	1.41	.164	0.51
Uniform sampling	Swan–Squirrel	6.2	32.7	− 19.06	< .001*	> 30
	Parrot–Goose	7.9 (18.0)	33.0 (32.0)	− 49.82	< .001*	> 30
	Whale–Snail	15.0	24.0	− 5.24	< .001*	> 30
	Duck–Rabbit	10.0 (15.8)	11.9 (12.0)	− 2.40	.020	2.64
	Swan–Cat	6.0	35.4	− 20.27	< .001*	> 30
Curvature-based	Swan–Squirrel	6.2	23.2	− 7.51	< .001*	> 30
	Parrot–Goose	7.9 (18.0)	25.7 (27.4)	− 8.48	< .001*	> 30
	Whale–Snail	15.0	24.9	− 3.89	< .001*	> 30
	Duck–Rabbit	10.0 (15.8)	24.1 (24.1)	− 5.92	< .001*	> 30
	Swan–Cat	6.0	23.4	− 8.18	< .001*	> 30
Combined semantic and curvature-based	Swan–Squirrel	6.2	4.9	NaN	NaN	NaN
	Parrot–Goose	7.9 (18.0)	11.3 (12.4)	4.79	< .001	> 30
	Whale–Snail	15.0	16.5	1.51	.137	0.58
	Duck–Rabbit	10.0 (15.8)	6.2 (6.6)	NaN	NaN	NaN
	Swan–Cat	6.0	7.1	2.13	.038	1.54

Significant *p* values are marked with an asterisk (Bonferroni-corrected significance level *p* < .010). Note that the combined semantic and curvature-based model is tested against the semantic organization model rather than against human data (NaNs follow from exactly the same values for both models).

**Figure 11:**
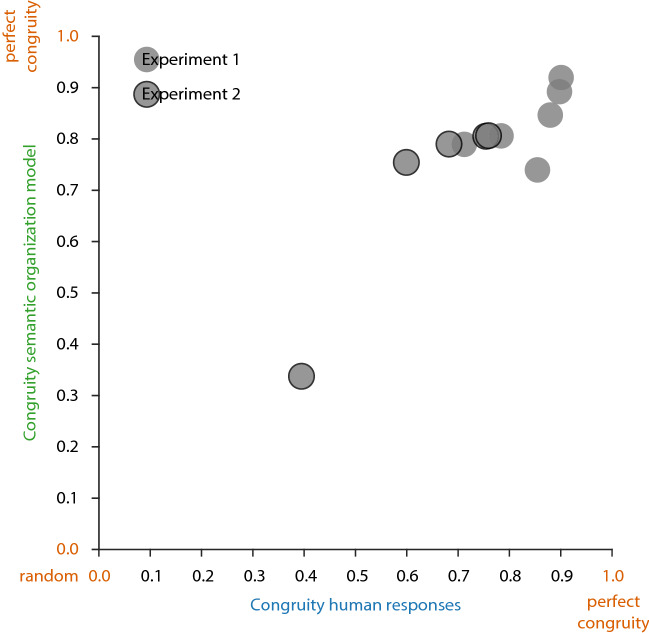
Congruity of the semantic organization model plotted against the congruity of human responses, separately for each stimulus pair in Experiments 1 and 2. As in Figs. 9 and 10, we plot response congruity on a continuum between random (0.0: congruity between responses of random model) and perfect congruity to human responses (1.0: all responses on top of each other).

Overall, this suggests that the semantic organization model is a good approximation of human behavior in both experiments (Fig. 11). In the following, we use the same distance metric to test plausible alternative models for predicting human responses.

### Testing plausible alternative models

First, we test a uniform sampling model which assumes that participants distribute their responses at equidistant intervals around the perimeter of the test shape, while replicating the order of the probe points. Second, we test a curvature-based model which assumes that participants choose corresponding points with respect to correspondences between curvature profiles of base and test shape^9^. Finally, we test whether the semantic organization model can be improved by including curvature-based information. To give the first two models the best possible chance, we first searched for the ‘starting point’ of the leftmost semantic part (e.g., tails in elephant and anteater; tail in parrot and head in goose) and re-sampled contour points in clockwise direction based on that starting position. Without doing that (e.g., if we would just sample from the leftmost point of every shape), predictions on curvature profiles (or uniform sampling) would be markedly less similar to human correspondence judgments—consequently, this represents a rather strict test to see whether the semantic organization model can better explain our data.

#### Uniform sampling model

**Figure 12:**
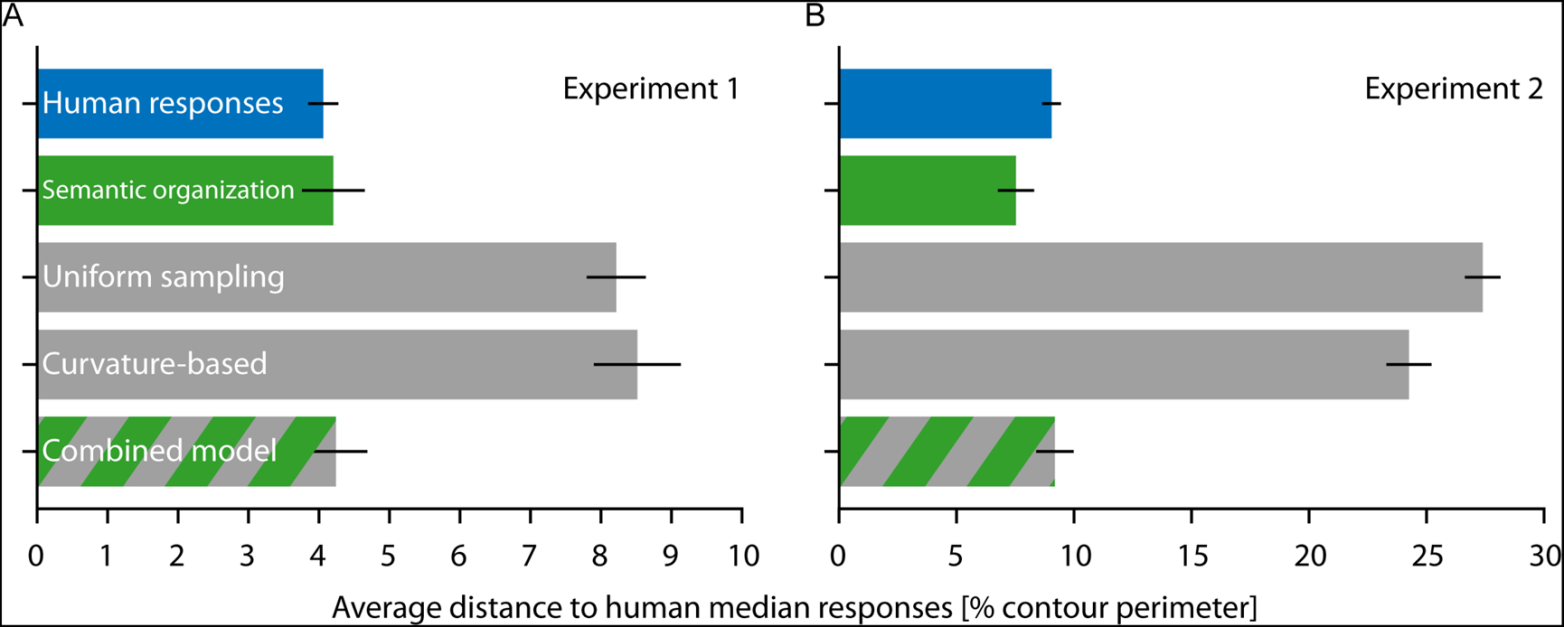
Average distances between human responses and predictions for (**A**) Experiment 1 and (**B**) Experiment 2. We plot the distance between the human response or model predictions and the human median response, expressed as percentage of the perimeter of the contour, and averaged across all stimulus pairs and sample points. The semantic organization model is well within the range of human responses, while the uniform sampling and curvature-based models perform much worse. The combined semantic organization plus curvature-based model does not yield better performance than the semantic organization model alone.

For Experiment 1, average distances of the uniform sampling model predictions to human median responses were 8.8%, 14.8%, 3.8%, 7.7%, 5.2% (13.6%), and 8.9% for the pairs (Table 1; grand average: Fig. 12). This was considerably lower than the distance between human and median responses (Table 1). To obtain predictions from the uniform sampling model for Experiment 2, we again inversed contour points because of the different heading directions of the two interpretations of each shape (Fig. 5A). The resulting average distances to human median responses were 32.7%, 33.0% (32.0%), 24.0%, 11.9% (12.0%), and 35.4% for the pairs (Table 2; grand average: Fig. 12), all of which were also considerably lower than distances between human and median responses (Table 2).

#### Curvature-based model

As in previous work^9^, we express contours in terms of their ‘surprisal’—an information theoretic measure, related to curvature, which quantifies how much each point on a contour ‘stands out’ with respect to its local neighborhood^34,35^. The basic assumption is that contours are likely to continue along their current tangent direction; and the more points on the contour diverge from this direction, the less predictable and therefore the more informative they are. This can be formalized by a continuous probability (*von Mises*) distribution on the turning angles centered on 0, which is producing monotonically decreasing probabilities (p) with increasing divergence from the current tangent direction. Surprisal is then formalized as u =  − log(p), increasing with turning angle^34^. Based on previous work, we calculated turning angles using a window integration size of 5% of the contour perimeter^9,10^, treated positive and negative curvature (i.e., convex and concave contour segments) symmetrically (*unsigned*;^35,36^) and normalized the surprisal for each point on a contour with respect to the maximum surprisal on that contour. In contrast to previous work, where we had to refer to ground truth transformations to find corresponding salient landmarks between base and test shapes^9^, ground truth is not available for the current stimulus set. Therefore, we use MATLAB’s (The MathWorks, Inc., Natick, Massachusetts, United States) dynamic time warping algorithm (e.g.,^37^) to find the optimal alignment between contour surprisal profiles of the base and test shapes based on their Euclidean distance. We use this alignment to project probe points from the base shape to the corresponding locations on the test shape. For Experiment 1, these predictions are pretty similar to human responses for the pairs of Elephant–Anteater and Lama–Fox, but much less for the other pairs: average distances of the curvature-based model predictions to human median responses were 1.4%, 16.7%, 6.2%, 3.0%, 8.9% (17.0%), and 15.0% (Table 1; grand average: Fig. 12). As a result, this was significantly lower than human congruity for four of the six pairs (Table 1). For Experiment 2, average distances to median responses were considerably higher: 23.2%, 25.7% (27.4%), 24.9%, 24.1% (24.1%), 23.4% (Table 2; grand average: Fig. 12). Overall, this shows that the curvature-based model is not a good fit for the human data, in contrast to previous experiments in which we tested shapes that were more similar or novel (i.e., with little semantic meaning;^9^).

#### Combined semantic organization plus curvature-based model

To test whether the semantic organization model can be improved by adding curvature information, we tested a model where predictions within each semantic part were distributed relative to salient landmarks within that part. For example, predictions for probe points on the trunk of the elephant would all be placed onto the snout of the anteater, but their exact position would depend on their relative position with respect to salient landmarks of both parts (e.g., their tips). We used the method established in^9^ to identify salient landmarks by (i) calculating the normalized distribution of surprisal values along the contour of the base shape (− 1, 1) and (ii) finding local minima/maxima that are surrounded by values that are higher/lower by 0.05 on both sides and have absolute values > 0.02. If the two corresponding semantic parts contained different numbers of local maxima and no unequivocal assignment was possible, we used the predictions from the semantic organization model. If the two corresponding semantic parts contained the same number of local maxima, and therefore allowed for an unequivocal assignment, we used the relative distance to those landmarks to predict the position of responses (with different numbers of local maxima, we kept the prediction from the semantic organization model).

For Experiment 1, the average distances of model predictions to human median responses were about the same as those of the semantic organization model (3.1%, 6.5%, 2.2%, 4.0%, 4.9% (12.1%), and 4.9%; Table 1; grand average: Fig. 12), when directly comparing the two models only performance for the Lizard-Whale pair was slightly better (Table 1). This demonstrates that for all pairs tested in Experiment 1, the semantic organization model could not be meaningfully improved by adding curvature-based information. For Experiment 2, responses were again very similar to those of the semantic organization model (4.9%, 11.3% (12.4%), 16.5%, 6.2% (6.6%), and 7.1%; Table 2; grand average: Fig. 12); when testing the two models against each other, the only significant difference was a poorer performance for the Parrot–Goose pair (Table 2).

Together, this shows that curvature information could not significantly improve the semantic organization model in explaining human responses—at least when considering our implementation of a combined model, and the shapes used in the current experiments.

## General discussion

Object shape is arguably the most important cue for object recognition and concept learning^4–8^. Here, we investigate our striking ability to identify point-to-point correspondences between object shapes (e.g.,^18–21^) with a focus on the contribution of cognitive processing (i.e., effects of previous knowledge about semantic organization). What is the role of shape correspondence in visual perception and cognition?

### Perceptual and cognitive functions of point-to-point correspondence

First, we can use shape correspondence to generalize across classes and, for example, predict potential object behavior. Having established correspondence between one animal with a trunk (elephant) and one with a long snout (anteater), or between two animals with pincers (crab and scorpion), we can make informed inferences about joint location and limb flexibility that will help us to predict animal or limb motion trajectories. More broadly, inferences based on these correspondences can potentially inspire new innovations; such as robot arms inspired by snakes or elephant trunks^38^. Second, establishing correspondence between different retinal projections of the same or similar object helps in preserving object constancy—the ability to identify objects across diverse viewing conditions or organisms across growth. For example, depending on viewing angle, an elephant’s trunk might be visible or not; depending on the age of an elephant, the elephant’s head will be larger or smaller compared to the body^39^.

Taking semantic organization into account, shape correspondence can facilitate and refine the organization of objects in a similarity space. For example, if two animals both have a head, horns and four legs, comparisons between those parts of the body will help us to decide how similar (i.e., closely related) the animals are: by comparing the shape of the head, horns and legs, we will tend to infer that a cow is more closely related to a bison than to a rhino^40^. Finally, we can also use correspondences to build sparse memory representations. By storing relevant information about an animal in terms of its main semantic parts together with its salient shape features, we can identify members of this animal class, or generate (e.g., imagine) new members of the same class (e.g., drawing an elephant to be recognized by others should include a bulky body, plump legs, flapping ears, a curved trunk and a narrow tail;^41,42^).

### Relative importance of semantics and geometrical features

Here, we were interested in the particular role of semantic organization in establishing correspondences. In previous work^9^, we showed that observers’ point-to-point correspondences across object transformations (such as rotation or growth) of unfamiliar objects were well explained by a model based on shape—with corresponding locations chosen relative to corresponding salient shape features on both shapes. In the current paper, we used stimuli designed to impede the potential of shape to guide correspondence judgments. The first experiment employed objects of very different shapes but similar semantic part organization; the second experiment employed objects of identical shape but different semantic part organization (where a response strategy based purely on shape would simply replicate all probe points on the test shape).

In line with our hypotheses and in contrast to previous work, we show that (i) observers agree with each other in establishing point-to-point correspondence between very different objects—suggesting that they follow the same or similar strategies; (ii) responses are affected by semantic part organization—which is most obvious in Experiment 2 where correspondences follow semantic interpretation rather than shape. We introduce a model that extends our previous modeling by predicting corresponding locations on the test shape not relative to corresponding salient geometrical features of two shapes but relative to their corresponding semantic parts. And, indeed, for almost all tested shape pairs, our model predicts median human responses as well as individual human observers do. At the same time a model purely based on shape performs considerably worse.

Together this suggests that humans use a straightforward approach to establish correspondences between objects of very different shapes. When two objects are novel (i.e., unfamiliar) but similar—so that we can easily determine the transformation between them—we find corresponding locations relative to corresponding salient geometrical features^9^. However, when objects are not similar enough for that—but we identify familiar elements (e.g., different body parts or extremities of animals)—we find corresponding locations relative to those parts (as illustrated in the current experiments).

In many cases, humans will use a combination of both approaches, depending on the availability of cues—that is, on the similarity of salient shape features^9,10^ or on the extent of knowledge about semantic part organization (Experiments 1 and 2). Accordingly, for unfamiliar shapes they would rather base their judgments on salient shape features; for familiar shapes with (sufficiently) similar part organization they would rather base their judgments on semantic information. As our stimuli were specifically designed to impede a strategy based on shape, we did not find any additional explanatory power of a model combining semantic part organization and shape information.

### Application to shape morphing

**Figure 13:**
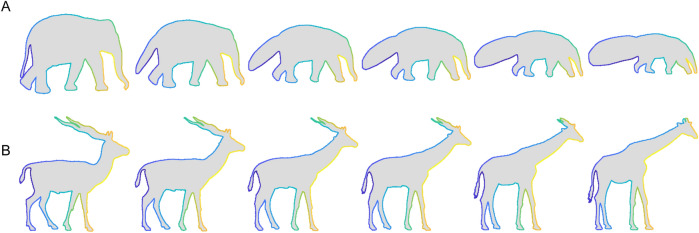
Examples of morphing based on the correspondence predictions of the semantic organization model for (**A**) Elephant–Anteater and (**B**) Antelope–Giraffe pairs of Experiment 1. Morphs are based on correspondences predicted by the semantic organization model with linear interpolation of in-between contour segments. Contours are colored according to their correspondence across morph levels. To avoid intersections, before building the morphs we excluded all predicted points on the test shape that showed order reversals with respect to the corresponding probe points on the base shape. Note that this straightforward approach will work less well for shapes with holes (e.g., Ostrich—Flamingo pair) or many order reversals (e.g., Lizard—Whale pair); for generalization the morphing procedure would need to be adapted.

A promising avenue for future research is the combination of our model with recent advances in machine learning. The latest deep neural networks trained on image segmentation potentially provide pixel-by-pixel semantic labels^43,44^. Through hierarchical segmentation, this information could be translated into semantic part organization (i.e., by identifying overlapping image regions corresponding to ‘bird’, ‘leg’ and ‘wing’). This information could be used to predict point-to-point correspondences on a large scale^45^; and also to morph objects into each other in a fashion in accordance with human perception. Figuring out perceptually sensible morphs without detailed user input (i.e., manual definition of anchor points in both objects), has been an endeavor in computer graphics and image processing for a long time (e.g.,^46,47^). As a proof of concept, we show how our model can be used to create perceptually sensible morphs between shapes with known semantic part organization. In Fig. 13, we show resulting morph examples for two of our shape pairs from Experiment 1.

In the future, such models combined with machine learning techniques will allow for advanced and potentially more human-like descriptions of objects and their shape. Current computer vision models compare shapes in terms of their geometric attributes (often in combination with manual user input, e.g.,^48^) and objects in terms of their texture properties (e.g.,^7^). In addition to these attributes, however, human observers also compare objects and shapes in terms of their deeper generative aspects, like their semantic attributes. Thus, to achieve more sophisticated and potentially human-like comparisons between shapes, we need models that incorporate a lexicon of simple geometric transformations (e.g., ‘rotate’, ‘stretch’, ‘shorten’, ‘bend’, ‘bloat’, ‘shrink’ and ‘enlarge’) with corresponding semantic labels (e.g., neck, legs, tail, body and horns). Such models would be able to compare two shapes by the differences between their corresponding parts. For example, the difference between an antelope and a giraffe might be summarized by a ‘stretching’ of neck, legs and tail, and a ‘shortening’ of body and horns (Fig. 2C). Thus, by identifying those transformations from the lexicon describing the relationship between two parts best (i.e., produces the smallest error), models will be able to describe more complex, non-linear transformations between objects in terms of simple transformations of their parts. Whether this leads to more human-like complex shape judgments by bringing together inferences about correspondence across transformations^9,10^ with those about past transformations from visual depictions of objects (*causal history*; e.g.,^49–54^) is an interesting area for future investigation.

### Limitations and future directions

As our model depends on the detail as well as on the quality of the available semantic part information, both will affect the accuracy of the resulting predictions. For example, it is not clear at what level of detail observers are typically operating (e.g., ‘Headʼ versus a distinction between ‘Headʼ, ‘Earsʼ and ‘Mouthʼ). Presumably, that level varies between participants but also between different stimuli or stimulus pairs. For example, if only one of the two shapes exhibit a particular semantic feature (e.g., ‘Earsʼ), observers might simply ignore them when building correspondences.

Our model cannot predict ambiguities and inter-individual differences in the interpretation of object shapes. For example, the wings of the Butterfly–Owl or the Parrot–Goose pair are ambiguous in their orientation (or viewing direction) and therefore ambiguous in their correspondence. Similarly, the rabbit in the Duck–Rabbit pair is ambiguous in its direction of heading (downwards versus right).

One way to resolve these ambiguities would be to simultaneously consider different predictions, with their probability derived from the frequency of the different semantic labeling responses. This might also help to resolve cases with less clear correspondences between semantic parts, for example, when asking participants to match locations on the four legs of a dog to a two-legged ostrich.

Another way to resolve these ambiguities would be to collect data and model predictions for 3D shapes rather than 2D contours. Of course, this would be technically more challenging—in terms of the dot matching procedure as well as in terms of the modeling. For example, a good model for predicting corresponding locations on a 3D surface would have to consider all ‘surrounding’ semantic parts of a particular probe point (e.g., ‘the probe location on the bird is slightly above the longitudinal axis of its right wing, halfway between head and tip of the tail’). Yet it is highly likely that observers can indeed exploit semantics to identify such correspondences in 3D.

Another avenue for this work is as a potential tool to reveal the interplay between perceptual and cognitive processes in bidirectional hierarchical neural networks. One might expect cognitive processes to be spatially imprecise and operate on abstract representations. However, our findings illustrate how humans rely flexibly on shape or semantic information in establishing local physical correspondence. Specifically, by solving the dot matching task, bottom-up perceptual organization is combined with top-down cognitive processes. How this is theoretically combined in bidirectional neural networks is still an open question. In terms of neural mechanisms, there has long been speculations about forward and backward pathways in the cortex (e.g.,^55–58^). Murray et al.^59^, for example, used fMRI to show that when local visual information is perceptually organized into whole objects, activity in lower areas (e.g., V1) decreases over the same period that activity in higher areas (e.g., lateral occipital cortex or LOC) increases. The results were interpreted in terms of high-level hypotheses that compete to explain away the lower-level retinal information. In the same manner, the edges and contours of shapes are activated in lower layers based on retinal information, and our higher-level cognitions (i.e., semantic judgements) can turn down the activity of these lower areas by explaining away the causal factors of these edges at the moment of perception. In effect, the present methods can be used to test the role of how such cognitive processes are merged with perception in the brain.

Finally, our findings are another building block in the description of *shape understanding* (e.g.,^60,61^): when we look at an object, we not only work out what shape it has, but also how or why it has that shape by parsing and interpreting its geometrical structure to identify its most important features and their relations to one another and to features of other objects. In the current work, we highlighted the interpretation component of *shape understanding* by biasing participants towards higher-level cognitive processes when establishing correspondences.

## Data Availability

The stimuli and raw data of the current study are available at 10.5281/zenodo.4304299.

## References

[1] Lake BM, Salakhutdinov R, Tenenbaum JB (2013). One-shot learning by inverting a compositional causal process. Adv. Neural Inf. Process Syst..

[2] Lake BM, Salakhutdinov R, Tenenbaum JB (2015). Human-level concept learning through probabilistic program induction. Science.

[3] Fei-Fei L, Fergus R, Perona P (2006). One-shot learning of object categories. IEEE Trans. Pattern Anal. Mach. Intell..

[4] Biederman I (1987). Recognition-by-components. A theory of human image understanding. Psychol. Rev..

[5] Morgenstern Y, Schmidt F, Fleming RW (2019). One-shot categorization of novel object classes in humans. Vis. Res..

[6] Landau B, Smith L, Jones S (1998). Object shape, object function, and object name. J. Mem. Lang..

[7] Geirhos, R. *et al.* ImageNet-trained CNNs are biased towards texture; increasing shape bias improves accuracy and robustness. *International Conference on Learning Representations*; https://openreview.net/forum?id=Bygh9j09KX (2019).

[8] Samuelson LK, Smith LB (2005). They call it like they see it. Spontaneous naming and attention to shape. Dev. Sci..

[9] Schmidt F, Fleming RW (2016). Visual perception of complex shape-transforming processes. Cogn. Psychol..

[10] Schmidt F, Spröte P, Fleming RW (2016). Perception of shape and space across rigid transformations. Vis. Res..

[11] Hahn U, Chater N, Richardson LB (2003). Similarity as transformation. Cognition.

[12] Hahn U, Close J, Graf M (2009). Transformation direction influences shape-similarity judgments. Psychol. Sci..

[13] Imai S (1977). Pattern similarity and cognitive transformations. Acta Psychol..

[14] Kimia BB, Tannenbaum AR, Zucker SW (1995). Shapes, shocks, and deformations I. The components of two-dimensional shape and the reaction-diffusion space. Int. J. Comput. Vis..

[15] Kubilius J, Bracci S, Op de Beeck HP (2016). Deep neural networks as a computational model for human shape sensitivity. PLoS Comput. Biol..

[16] Ons B, Wagemans J (2012). Generalization of visual shapes by flexible and simple rules. Seeing Perceiv..

[17] Panis S, Vangeneugden J, Wagemans J (2008). Similarity, typicality, and category-level matching of morphed outlines of everyday objects. Perception.

[18] Moran S, Leiser D (2002). The limits of shape constancy. Point-to-point mapping of perspective projections of flat figures. Behav. Inf. Technol..

[19] Phillips F, Todd JT, Koenderink JJ, Kappers AML (1997). Perceptual localization of surface position. Exp. Psychol. Hum. Percept. Perform..

[20] Phillips F, Todd JT, Koenderink JJ, Kappers AML (2003). Perceptual representation of visible surfaces. Percept. Psychophys..

[21] Koenderink JJ, Kappers AM, Pollick FE, Kawato M (1997). Correspondence in pictorial space. Percept. Psychophys..

[22] Koenderink JJ, van Doorn AJ, Kappers AML, Todd JT (1997). The visual contour in depth. Percept. Psychophys..

[23] Hoffman DD, Richards WA (1984). Parts of recognition. Cognition.

[24] Hummel JE, Biederman I (1992). Dynamic binding in a neural network for shape recognition. Psychol. Rev..

[25] Siddiqi K, Tresness KJ, Kimia BB (1996). Parts of visual form psychophysical aspects. Perception.

[26] Kleiner M, Brainard D, Pelli D (2007). What’s new in psychtoolbox-3?. Perception.

[27] Fisher GH (1968). Ambiguity of form: old and new. Percept. Psychophys..

[28] Tinbergen N (1951). The Study of Instinct.

[29] Bernstein LJ, Cooper LA (1997). Direction of motion influences perceptual identification of ambiguous figures. Exp. Psychol. Hum. Percept. Perform..

[30] Jastrow J (1900). Fact and Fable in Psychology.

[31] De Winter J, Wagemans J (2006). Segmentation of object outlines into parts. A large-scale integrative study. Cognition.

[32] Rouder JN, Speckman PL, Sun D, Morey RD, Iverson G (2009). Bayesian t tests for accepting and rejecting the null hypothesis. Psychon. Bull. Rev..

[33] Jeffreys H (1961). Theory of Probability.

[34] Feldman J, Singh M (2005). Information along contours and object boundaries. Psychol. Rev..

[35] Attneave F (1954). Some informational aspects of visual perception. Psychol. Rev..

[36] Norman JF, Phillips F, Ross HE (2001). Information concentration along the boundary contours of naturally shaped solid objects. Perception.

[37] Paliwal KK, Agarwal A, Sinha SS (1982). A modification over Sakoe and Chiba’s dynamic time warping algorithm for isolated word recognition. Signal. Process..

[38] Webster RJ, Jones BA (2010). Design and kinematic modeling of constant curvature continuum robots. A review. Int. J. Robot..

[39] Todd JT, Mark LS, Shaw RE, Pittenger JB (1980). The perception of human growth. Sci. Am..

[40] Ohl M, Henke W, Tattersall I (2007). Principles of taxonomy and classification: current procedures for naming and classifying organisms. Handbook of Paleoanthropology.

[41] Mukherjee, K., Hawkins, R. & Fan, J. Communicating semantic part information in drawings. In *CogSci 2019* (eds. Goel, A., Seifert, C. & Freksa, C.) 1–7 (2019).

[42] Tiedemann H, Morgenstern Y, Schmidt F, Fleming RW (2019). Novel object categories generated from single exemplars. Perception.

[43] Liu X, Deng Z, Yang Y (2019). Recent progress in semantic image segmentation. Artif. Intell. Rev..

[44] Huang, S., Xu, Z., Tao, D. & Zhang, Y. Part-stacked CNN for fine-grained visual categorization. *Proc. IEEE Comput. Soc. Conf. Comput. Vis. Pattern Recognit.* 1173–1182 (2016).

[45] Dyke RM (2020). SHREC’20: shape correspondence with non-isometric deformations. Comput. Gr..

[46] Yang W, Feng J (2009). 2D shape morphing via automatic feature matching and hierarchical interpolation. Comput. Gr..

[47] Beier T, Neely S (1992). Feature-based image metamorphosis. Comput. Gr..

[48] Zuffi, S., Kanazawa, A., Jacobs, D. & Black, M. J. 3D Menagerie: modeling the 3D Shape and Pose of Animals. *Proc. IEEE Comput. Soc. Conf. Comput. Vis. Pattern Recognit.* 5524–553 (2017).

[49] Leyton M (1989). Inferring causal history from shape. Cogn. Sci..

[50] Arnheim R (1974). Art and Visual Perception: A Psychology of the Creative Eye.

[51] Pinna B (2010). New Gestalt principles of perceptual organization: an extension from grouping to shape and meaning. Gestalt Theory.

[52] Fleming RW, Schmidt F (2019). Getting, “fumpered”. Classifying objects by what has been done to them. J. Vis..

[53] Schmidt F, Phillips F, Fleming RW (2019). Visual perception of shape-transforming processes. ‘Shape Scission’. Cognition.

[54] Schmidt F, Fleming RW (2018). Identifying shape transformations from photographs of real objects. PLoS ONE.

[55] Hochstein S, Ahissar M (2002). View from the top: hierarchies and reverse hierarchies in the visual system. Neuron.

[56] Mumford D (1992). On the computational architecture of the neocortex. II. The role of cortico-cortical loops. Biol. Cybern..

[57] Kersten D, Yuille AL, Werner JS, Chalupa LM (2013). Vision: bayesian inference and beyond. Vision: Bayesian Inference and Beyond The New Visual Neurosciences.

[58] Yuille A, Kersten D (2006). Vision as Bayesian inference: analysis by synthesis?. Trends Cogn. Sci..

[59] Murray SO, Kersten D, Olshausen BA, Schrater P, Woods DL (2002). Shape perception reduces activity in human primary visual cortex. Proc. Natl. Acad. Sci. USA.

[60] Spröte P, Schmidt F, Fleming RW (2016). Visual perception of shape altered by inferred causal history. Sci. Rep..

[61] Pinna B, Koenderink J, van Doorn A (2015). The phenomenology of the invisible. From visual syntax to “shape from shapes”. Philos. Sci..

